# Cue-induced cocaine craving enhances psychosocial stress and vice versa in chronic cocaine users

**DOI:** 10.1038/s41398-022-02204-5

**Published:** 2022-10-11

**Authors:** Ann-Kathrin Kexel, Bruno Kluwe-Schiavon, Markus R. Baumgartner, Etna J. E. Engeli, Monika Visentini, Clemens Kirschbaum, Erich Seifritz, Beate Ditzen, Leila M. Soravia, Boris B. Quednow

**Affiliations:** 1 Experimental and Clinical Pharmacopsychology, Department of Psychiatry, Psychotherapy, and Psychosomatics, Psychiatric University Hospital, University of Zurich, Lenggstrasse 31, 8032 Zurich, Switzerland; 2 Cognition in Context, Research Center for Psychological Science, University of Lisbon, Alameda da Universidade, 1649-013 Lisbon, Portugal; 3 Centre for Forensic Hair Analytics, Institute of Forensic Medicine, University of Zurich, Kurvenstrasse 17, 8006 Zurich, Switzerland; 4 Centre for Addictive Disorders, Department of Psychiatry, Psychotherapy, and Psychosomatics, Psychiatric University Hospital, University of Zurich, Selnaustrasse 9, 8001 Zurich, Switzerland; 5 Department of Biopsychology, Technical University Dresden, Zellescher Weg 19, 01069 Dresden, Germany; 6 Department of Psychiatry, Psychotherapy, and Psychosomatics, Psychiatric University Hospital, University of Zurich, Lenggstrasse 31, 8032 Zurich, Switzerland; 7 Neuroscience Center Zurich, University of Zurich and Swiss Federal Institute of Technology Zurich, Winterthurerstrasse 190, 8057 Zurich, Switzerland; 8 Institute for Medical Psychology, Heidelberg University Hospital, Heidelberg, Germany; 9 Ruprecht-Karls University Heidelberg, Bergheimer Str. 20, 69115 Heidelberg, Germany; 10 Translational Research Center, University Hospital of Psychiatry, University of Bern, Bolligenstrasse 111, 3000 Bern 60, Switzerland

**Keywords:** Addiction, Physiology, Psychology

## Abstract

Stress and craving, it has been found, contribute to the development and maintenance of and relapse in cocaine use disorder. Chronic cocaine users (CU), previous research has shown, display altered physiological responses to psychosocial stress and increased vegetative responding to substance-related cues. However, how psychosocial stress and cue-induced craving interact in relation to the CU’s physiological responses remains largely unknown. We thus investigated the interaction between acute psychosocial stress and cocaine-cue-related reactivity in 47 CU and 38 controls. In a crossed and balanced design, the participants were randomly exposed to a video-based cocaine-cue paradigm and the Trier Social Stress Test (TSST) or vice versa to investigate possible mutually augmenting effects of both stressors on physiological stress responses. Over the course of the experimental procedure, plasma cortisol, ACTH, noradrenaline, subjective stress, and craving were assessed repeatedly. To estimate the responses during the cocaine-cue paradigm and TSST, growth models and discontinuous growth models were used. Overall, though both groups did not differ in their endocrinological responses to the TSST, CU displayed lower ACTH levels at baseline. The TSST did not elevate craving in CU, but when the cocaine-cue video was shown first, CU displayed an enhanced cortisol response to the subsequent TSST. In CU, cocaine-cues robustly evoked craving but no physiological stress response, while cue-induced craving was intensified after the TSST. Taken together, though CU did not show an altered acute stress response during the TSST, stress and craving together seemed to have mutually augmenting effects on their stress response.

## Introduction

Stress has been repeatedly proposed to critically impact the development and maintenance of and relapse in substance use disorders, such as cocaine use disorder (CUD) [1–3]. Accordingly, strong overlaps exist between reward- and stress-related neurocircuits that interact during stress and drug use [1, 3, 4]. With continued substance use, adaptations occur in these neurocircuits that alter drugs’ rewarding effects and the motivation to use them. Moreover, maladaptive stress responses are enhanced, contributing to compulsive drug use and continued relapse vulnerability even a long time after the cessation of substance use [3, 5, 6].

As a regulator of physiological stress responses, the hypothalamic–pituitary–adrenal (HPA) axis has received broad attention in the CUD context. Acute cocaine administration induces an increase in adrenocorticotropin (ACTH) and cortisol secretion [7–9] in humans, suggesting a HPA-axis’ cocaine-induced stimulation. The cocaine-induced stimulation of the HPA-axis, it has been proposed, is mediated by hypothalamic corticotropin releasing factor [10–13]. As per animal models, the HPA-axis activation is involved in the acquisition, maintenance, and reinstatement of cocaine self-administration [14, 15]. The HPA-axis activity thus heightens the sensitivity for cocaine reward and influences individuals’ susceptibility to develop CUD [14]. Moreover, chronic cocaine administration, animal studies indicated, augments the physiological stress load and changes HPA-axis reactivity over time [5, 16, 17]. Accordingly, hospitalized cocaine users (CU) showed elevated plasma [18, 19] and salivary cortisol levels [20]. Chronic CU exhibit lower glucocorticoid receptor gene (NR3C1) expression in blood [21], and as per the longitudinal analysis of this sample, CU’s NR3C1 expression normalized with reduced cocaine consumption [22]. Specifically, a dysregulated HPA-axis response was suggested to increase the relapse probability due to substance use’s negative reinforcement properties [2, 3, 5, 23]. Accordingly, a blunted salivary cortisol response was observed in CU and methamphetamine users during the Trier Social Stress Test (TSST) and personalized stress imagery [24], whereas another study observed only a blunted plasma cortisol response to the TSST in female CU [25]. Longitudinal studies have linked the HPA-axis response to laboratory-induced stress with later relapse in dependent CU. Increased cortisol reactivity in a personalized stress-related imagery-task was related to greater cocaine use during follow-up [26], whereas Back et al. [27] reported that a blunted ACTH and cortisol reaction to the TSST are predictive of cocaine use and a shorter relapse time. Although both a blunted and hyper-responsive HPA-axis are indicative of a dysregulated stress response [3], these previous results highlight the heterogeneity of the present findings in this field. Furthermore, most stress studies had only relatively small sample sizes, did not include healthy control groups, and mainly relied on subjective reports of substance use.

Craving is also associated with a greater relapse susceptibility in CUD [3, 4, 6, 23]. For CU, exposure to experimental stress and drug-related cues evokes similar responses in the HPA- and sympathetic-adrenal medullary (SAM) axis and induces craving and subjective stress [25, 28–30]. Real-life stress is also associated with craving in cocaine- and heroin-dependent outpatients [31]. A higher frequency of cocaine and alcohol use led to (1) stronger craving in response to a personalized stress-related imagery-task, and (2) stronger craving and a greater HPA-axis reactivity in response to a personalized drug-related imagery-task, suggesting that more intense substance use increases individuals’ proneness to relapse by heightening their vulnerability to stress and drug paraphernalia [32]. Longitudinal studies have associated the subjective response to laboratory-induced stress and cocaine-related cues with later relapse. Increased cocaine craving in a personalized stress-related imagery-task [26] and greater cue-induced craving and subjective stress were related to a shorter relapse time [27].

Although stress and craving seem to trigger cocaine use, how they interact remains unknown. To our knowledge, no study has yet tried to disentangle these effects and investigated the influence of a preceding cocaine-cue on subsequent psychosocial stress reactivity. Though the effect of preceding psychosocial stress on subsequent drug-cue reactivity has been examined in a number of substances [33–37], research on chronic CU is lacking. Since previous research separately investigated stress and craving in CU, studying their combined influence—stress and craving should influence each other in everyday life—is the next step in increasing the everyday validity of results. Considering that (1) the HPA-axis activity increases cocaine reward sensitivity and is involved in cue-induced cocaine reinstatement in animal models [14, 15, 38] and (2) stress activates the dopaminergic mesocorticolimbic reward system (for a review, see [5]), one might hypothesize that the cue-induced dopamine-mediated prediction error for cocaine reward is amplified by the preceding stimulation of the HPA-axis through psychosocial stress, leading to greater cocaine-cue reactivity. Accordingly, enhanced activation in brain areas associated with reward and conditioned cues were observed during cocaine-cue imagery when a stressor was present [39], and cocaine craving was exacerbated when real life stress and drug-cues were present simultaneously in opioid-dependent polydrug users [40]. Furthermore, as exposure to cocaine-cues can be considered stressful [25, 28–30], one might also assume an amplified psychosocial stress reactivity in CU when exposed to a cocaine-cue beforehand.

This study aimed to investigate acute psychosocial stress and cocaine-cue reactivity and their interaction in chronic CU whose cocaine use was objectively quantified by hair toxicology. The TSST, a motivated performance task with high levels of social-evaluative threat and uncontrollability [41], was used to induce psychosocial stress [42]. It reliably induces subjective and physiological stress responses in the HPA- and SAM-axis (for a review, see [43]) and may elicit craving in CU [25]. Craving and craving-induced stress were evoked with a video-based cocaine-cue paradigm of high ecological validity [44]. The TSST and the cocaine-cue paradigm were applied consecutively in a randomized, crossed, and balanced design in CU and stimulant-naïve healthy controls (HC) to examine possible augmenting effects of both stressors. Half of the participants therefore underwent the TSST first and the cocaine-cue paradigm second, whereas the other half underwent the cocaine-cue paradigm first and the TSST second. The stress and craving responses were analyzed using discontinuous and continuous growth models. Based on previous research, we hypothesized that (1) the TSST and cocaine-cue paradigm increase craving in CU; (2) psychosocial stress evokes HPA-axis responses, with a more blunted response in CU; (3) the cocaine-cue paradigm elicits HPA-axis responses only in CU; (4) psychosocial stress and cocaine-cue reactivity intensify each other.

## Methods

### Participants

In the context of the *Social Stress Cocaine Project* (SSCP) [45], 69 CU and 54 HC were recruited. In- and exclusion criteria were tested during a screening-session. General inclusion criteria were being able to read, understand, and provide written-informed consent; German fluency; age between 18–50. Specific inclusion criteria for chronic CU were an estimated cumulative lifetime cocaine consumption of >100 g; cocaine as the primary used illegal drug; current cocaine use or if abstinent, a current abstinence duration of <6 months. General exclusion criteria were a neurological disorder or brain injury; a current diagnosis of an infectious disease or severe somatic disorder; a history of an autoimmune, endocrine, and rheumatoid disease; intake of medication with potential action on the central nervous system, immune system, or HPA-axis during the last three days; a family history of genetically mediated psychiatric disorders (*h*^2^ > 0.5; e.g., autism spectrum disorder, bipolar disorder, and schizophrenia); participation in the *Zurich Cocaine Cognition Study*, a previous study from our group [46, 47]; for women, pregnancy, breastfeeding, or menstruation. Specific exclusion criteria for CU were opioid use disorder; current polysubstance use; DSM-IV-R Axis I adult psychiatric disorders except other substance use disorders, attention-deficit-hyperactivity disorder (ADHD), and previous depressive episodes. Specific exclusion criteria for HC were recurrent illegal substance use (>15 occasions lifetime, except cannabis use); DSM-IV-R Axis I adult psychiatric disorders. After applying these criteria and counting dropouts at the stress-session, a sample of 85 individuals (47 CU, 38 HC) was included in the data analysis (see Supplement). The sample size was determined by a priori power analysis with G*Power 3 [48]. In cross-sectional studies, dependent cocaine users and controls differed with large effect sizes (Cohen’s *d* = 0.71–0.83) regarding neuroendocrinological stress measures at baseline [19, 20]. Thus, assuming a large effect size, an *α*-error probability of 5% and a power estimation of 85% in a four-group design, we would need a total sample size of at least 59 individuals.

The Cantonal Ethics Committee of Zurich (ID 2016-00278) approved the study, which was preregistered in the *International Standard Randomized Controlled Trial Number Registry* (ISRCTN10690316). All participants provided written-informed consent under the Declaration of Helsinki.

### Clinical and substance use assessment

The psychopathological evaluation with the *Structured Clinical Interview-I* for DSM-IV Axis I disorders [49] was conducted at the screening-session (see Supplement for further questionnaires). The structured and standardized *Interview for Psychotropic Drug Consumption* [50] assessed self-reported substance use, which was objectively quantified by hair analyses of a proximal 4 cm hair segment (representing substance use during approximately four months prior to each assessment) using liquid chromatography tandem mass spectrometry [51] (LC-MS/MS). Individuals were asked to abstain from illegal drug use 72 h, from alcohol use 24 h and from caffeine intake two hours prior to the test sessions. To verify compliance with abstinence instructions, urine toxicology screenings with semi-quantitative enzyme multiplied immunoassays targeting amphetamines, barbiturates, benzodiazepines, cocaine, methadone, morphine-related opiates, and tetrahydrocannabinol were performed.

### Procedure and study design

The standard TSST includes a resting, preparation (10 min), test (10 min), and recovery period (for a detailed description of the TSST protocol, see [42, 52]) and reliably induces acute psychosocial stress (for a review, see [43]). Craving and related stress were induced using a video-based cocaine-cue paradigm (Cocaine-Cue-Video) [44]. Participants watched 10 min of a neutral scene and, subsequently, 10 min of a cocaine preparation and consumption scene (analogous to the TSST preparation and test period).

**Fig. 1:**
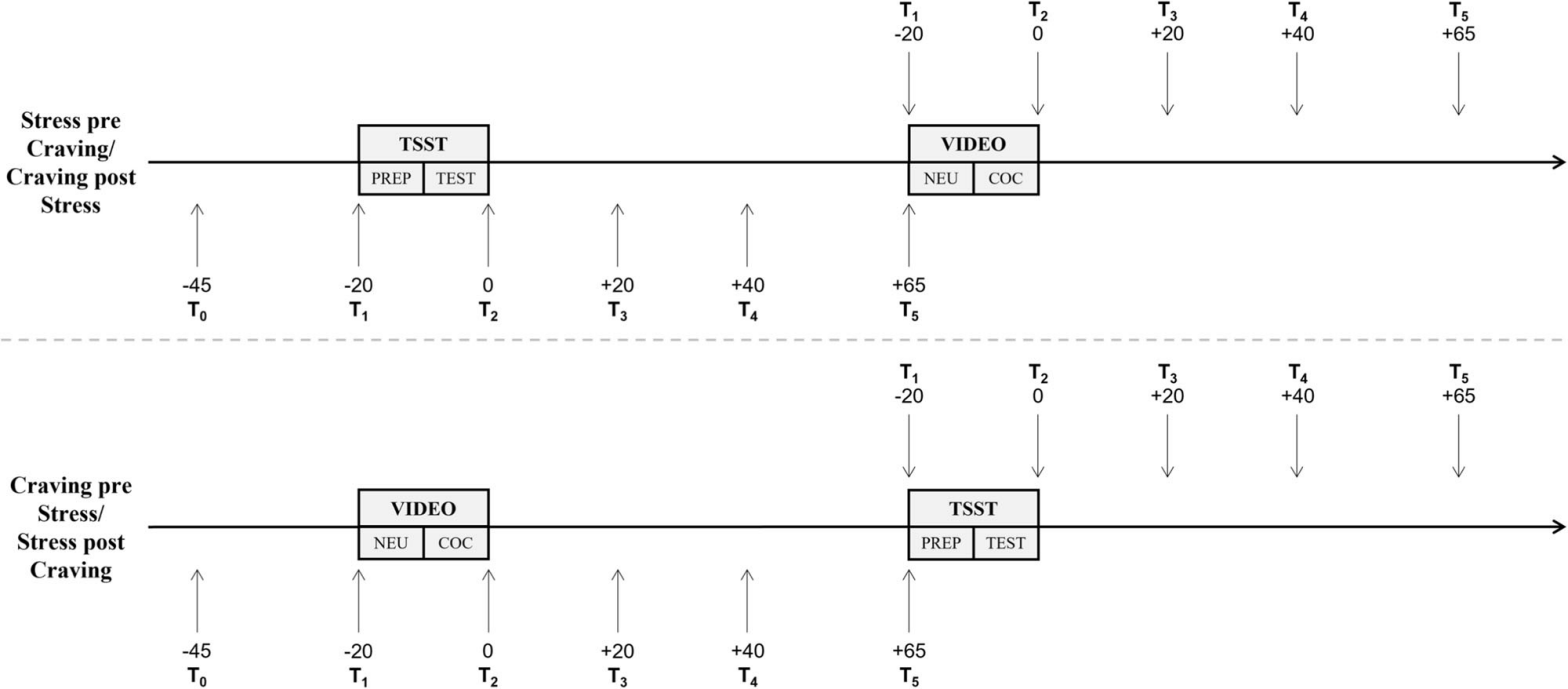
Schematic overview of the test session. PREP = TSST preparation period; TEST = TSST test period; NEU = Neutral Video; COC = Cocaine Video.

The baseline blood sample (*T*_0_) was taken between 01:00 p.m. and 02:15 p.m. The first stress challenge began 25 min later. The order of the stress challenges was crossed, and counterbalanced to evaluate how psychosocial and craving/craving-induced stress interact (Fig. 1). Simple randomization was used to assign CU and HC their respective order of the stress challenges while still controlling for relatively equal age and sex distribution across conditions. Half of the participants therefore received the TSST first and the Cocaine-Cue-Video second, whereas the other half received the Cocaine-Cue-Video first and the TSST second. The first stress challenge occurred during early afternoon (01:30 p.m. – 02:45 p.m.), and the second stress challenge during later afternoon (03:15 p.m. – 04:30 p.m.). For more details, see Supplement.

### Outcome measures

#### Neuroendocrine responses

Blood samples were drawn 20 min (*T*_1_) before the end of the TSST/Cocaine-Cue-Video as well as 0 (*T*_2_), 20 (*T*_3_), 40 (*T*_4_), and 65 min (*T*_5_) after the TSST/Cocaine-Cue-Video (Fig. 1) with BD Vacutainer^®^ EDTA-tubes that were immediately centrifuged. Plasma was aliquoted and stored at −80 °C until analysis. Samples from seven individuals (5 CU, 2 HC) were not taken due to problems with placing the i.v. catheter. ACTH (ACTH ELISA, IBL International GmbH, Hamburg, Germany) and cortisol (Cortisol ELISA, IBL International GmbH, Hamburg, Germany) were analyzed through enzyme-linked immunosorbent assays at Dresden LabService GmbH (Technical University of Dresden, Dresden, Germany). High-performance liquid chromatography was used to determine noradrenaline (ClinRep® HPLC Complete Kit, Recipe Chemicals + Instruments GmbH, Munich, Germany). Inter-assay coefficients of variation for cortisol were 7.2%, for ACTH 8.8%, and for noradrenaline 5.2%. Intra-assay coefficients of variation for cortisol were 3.5%, for ACTH 7.5%, and for noradrenaline 2.3%. The primary outcome measure was plasma cortisol. Salivary cortisol was also assessed, but the data are not shown since salivary and plasma cortisol levels were strongly correlated [53]. Six individuals had single time-point missing data (0.8%), three had missing data at two time-points (0.8%), and one at eight time-points (1.0%) in noradrenaline. The handling of missing data is explained in the Supplement.

#### Subjective stress and craving ratings

Subjective stress and craving were estimated with an 11-point Numeric Rating Scale (0 = not stressed/no craving, 10 = very stressed/high craving; with quarterly intervals) at the beginning of the test day (*T*_0_), directly before (*T*_1_), and after (*T*_2_) the TSST preparation period/Neutral-Video, and directly after the TSST test period/Cocaine-Video (*T*_3_), and 65 min later (*T*_4_).

### Statistical analysis

Demographic, clinical, and substance use data were analyzed with Pearson’s *χ*^2^-test, Student’s *t* test or, if the data were non-normally distributed or showed heterogeneity of variance, with Mann–Whitney *U*-test and Welch’s *t* test, respectively. Using Student’s *t* tests, the baseline group differences in neuroendocrine levels and subjective stress (*T*_0_) were identified. See Supplement for correlational analyses.

#### TSST and Cocaine-Cue-Video

##### Trajectories

Due to the hierarchical data structure, discontinuous growth models, a variation of linear mixed models (LMMs), were used to analyze the neuroendocrine, subjective stress, and craving response over the course of the TSST, and the subjective stress and craving response over the course of the Cocaine-Cue-Video. Based on the TSST stress response’s known trajectory (e.g., [43]) and the visual appearance of the descriptive trajectories, cortisol, noradrenaline, subjective stress, and craving were divided into three linear components and ACTH into four (for details and coding schemes, see the Supplement). To model the neuroendocrine response over the course of the Cocaine-Cue-Video, a linear (time) and quadratic (time^2^) time slope (centered on *T*_1_) were used in continuous growth models. Two-level models were fitted with individual samples (level-1) nested in individuals (level-2), including a random-intercept for participant ID. The time components and a group variable (dummy-coded with levels “CU-Stress-pre-Craving,” “CU-Stress-post-Craving,” “HC-Stress-pre-Craving,” “HC-Stress-post-Craving” for the TSST; with levels “CU-Craving-pre-Stress,” “CU-Craving-post-Stress,” “HC-Craving-pre-Stress,” “HC-Craving-post-Stress” for the Cocaine-Cue-Video) together with interactions between the time components and group were entered as fixed-effects to evaluate whether, depending on the onset of the experimental challenge, CU differed from HC and from each other. In the TSST analysis, the CU of the Stress-pre-Craving-group were defined as the reference group, and in the analysis of the Cocaine-Cue-Video, the CU of the Craving-pre-Stress-group were defined as the reference group. Before analysis, ACTH, noradrenaline, and craving were ln-transformed to approach normal distribution. Model assumptions were verified.

Since the respective dependent variable’s baseline (*T*_0_) levels improved model fit according to Bayesian Information Criterion (BIC), they were included as a covariate. For the noradrenaline trajectory during the Cocaine-Cue-Video, cannabis consumption was included for the same reason. The covariates—sex, age, BMI, verbal IQ, smoker, cannabis, MDMA, and alcohol consumption—did not improve model fit according to BIC, with the results remaining robust despite their influence and are thus unreported (for more information, see Supplement).

##### Area under the curve

Analyses of covariance (ANCOVAs) with the factors group (HC–CU) and order (for TSST: Stress-pre-Craving – Stress-post-Craving; for Cocaine-Cue-Video: Craving-pre-Stress – Craving-post-Stress) were used to establish differences in area under the curve with respect to ground (AUC_G_) (see: Supplement). AUC_G_ for ACTH, noradrenaline, and craving were ln-transformed to approach normal distribution. Levene’s test verified homogeneity of variance.

To assess interactions between the subjective and physiological stress response during the TSST, mixed ANOVAs with the between-subjects factors group and order and the within-subjects factor type-of-stress-response (subjective – physiological) were conducted on the z-transformed values of AUC_G_, which were sqrt-transformed in the analyses of ACTH and noradrenaline.

The data were analyzed using IBM SPSS Statistics 25.0 except LMMs, which were analyzed with the “nlme” package [54] in R [55] and fitted with maximum likelihood estimation. The significance level was *p* < 0.05 (two-sided). We consider trajectories and AUC_G_ for each outcome measure as separate constructs that do not represent the same information but rather measure a reaction in different systems. This is why we did not adjust for multiple comparisons.

## Results

### Demographic characteristics and substance use

Groups did not differ significantly in age, sex, smoking status, and cannabis lifetime experience (Table 1). However, CU had greater weekly alcohol and nicotine use, higher BMI, lower verbal IQ, fewer years of school education and, as expected, scored higher on the *Attention-Deficit/Hyperactivity-Disorder Self-Rating Scale* and the *Beck Depression Inventory* than HC. Moreover, CU’s self-reported substance use and hair toxicological results showed a clear cocaine preference (Tables 1 and S1). In all, 32% (*n* = 15) of CU reported being in treatment or counseling for cocaine use and 15% (*n* = 7) for other mental problems. Thus, 53% (*n* = 25) were currently not treated. CU used primarily powder cocaine and administered it intranasally.

**Table 1:** Demographic, clinical, and substance use-related data.

	Controls (*n* = 38)	Cocaine users (*n* = 47)	Test statistic	df	*p*
*Demographics*					
Sex (m/f) (*n*)	24/14	31/16	*χ*^2^ = 0.1^g^	1	0.788
Age	29.5 (7.1)	31.8 (7.7)	*t* = −1.4^h^	83	0.153
BMI	23.1 (3.2)	24.9 (3.8)	*t* = −2.3^h^	83	**0.021**
Verbal IQ	103 (9.2)	95.5 (6.1)	*t* = 4.1^i^	61.37	**<0.001**
Years of school education	10.5 (1.5)	9.6 (1.0)	*t* = 3.4^i^	62.61	**0.001**
*Clinical*					
ADHD-SR sum score	10.1 (9.6)	14.7 (10.3)	*t* = −2.1^h^	83	**0.036**
ADHD, y/n^a^	5/33	12/35	*χ*^2^ = 2.0^g^	1	0.156
BDI sum score^b^	1.0 (0.0–22.0)	7.0 (0.0–31.0)	*U* = 498^j^		**<0.001**
Treatment or counseling (*n*)					
Cocaine use		15			
Other		7			
*Nicotine*					
Smoker/non-smoker (*n*)^c^	29/9	37/10	*χ*^2^ = 0.1^g^	1	0.791
Cigarettes/week^c,d,e^	70.0 (7.0–158)	90.0 (33.3–280)	*U* = 339^j^		**0.010**
Years of use^d^	12.2 (6.4)	15.6 (7.3)	*t* = −2.0^h^	64	0.053
*Alcohol*					
Times/week^c,e^	2.1 (0.0–8.5)	2.0 (0.0–11.0)	*U* = 889^j^		0.972
Grams/week^c,e^	45.8 (0.4–248)	100 (0.0–672)	*U* = 646^j^		**0.029**
Years of use	14.4 (6.9)	16.6 (7.3)	*t* = −1.4^h^	83	0.155
Abstinence (days)^c^	4.0 (1.0–137)	5.00 (0.0–248)	*U* = 889^j^		0.972
*Cannabis*					
Lifetime experience, y/n	31/7	43/4	*χ*^2^ = 1.8^g^	1	0.176
Times/week^c,e^	0.0 (0.0–2.0)	0.1 (0.0–6.0)	*U* = 657^j^		**0.028**
Grams/week^c,e^	0.0 (0.0–0.6)	0.0 (0.0–12.6)	*U* = 663^j^		**0.032**
Years of use	6.2 (6.0)	11.5 (9.7)	*t* = −3.1^i^	78.27	**0.003**
Abstinence (days)^c^	163 (2.0–8807)	51.0 (1.0–10,753)	*U* = 565^j^		**0.266**
Cumulative lifetime dose (grams)^c^	3.5 (0.0–1972)	426.3 (0.0–25,719)	*U* = 472^j^		**<0.001**
THC, pg/mg in hair^c^	0.1 (0.0–320)	2.0 (0.0–540)	*U* = 687^j^		0.057
CBN, pg/mg in hair^c^	0.0 (0.0–47.0)	0.0 (0.0–170)	*U* = 621^j^		**0.003**
CBD, pg/mg in hair^c^	0.0 (0.0–26.0)	0.6 (0.0–75.0)	*U* = 636^j^		**0.018**
Urine toxicology (neg/pos)	36/2	41/6	*χ*^2^ = 1.8^g^		0.239
*Cocaine*					
Lifetime experience, y/n	5/33	47/0			
Times/week^e^		2.4 (2.2)			
Grams/week^e^		4.0 (6.7)			
Route of administration (*n*)					
Intranasal		44			
Intravenous		1			
Smoking		2			
Years of use		12.1 (7.4)			
Abstinence (days)		26.3 (46.3)			
Cumulative lifetime dose (grams)		1750 (2145)			
Cocaine_total_, pg/mg in hair^f^		24,703 (59,913)			
Cocaine, pg/mg in hair		18,438 (44,527)			
Benzoylecgonine, pg/mg in hair		5799 (15,046)			
Norcocaine, pg/mg in hair		467 (882)			
Cocaethylene, pg/mg in hair		512 (861)			
Urine toxicology (neg/pos)	38/0	26/21	*χ*^2^ = 22.5^g^	1	**<0.001**
Cocaine dependency current, y/n	0/38	31/16	*χ*^2^ = 39.5^g^		**<0.001**
Cocaine dependency past, y/n	0/38	33/14	*χ*^2^ = 43.6^g^		**<0.001**

Significant *p* values are shown in bold. Counts or means and standard deviation of means in parenthesis.

*ADHD-SR* ADHD self-rating scale, *BDI* Beck Depression Inventory.

^a^According to DSM-IV criteria as assessed by ADHD-SR.

^b^Median (range) is reported.

^c^Individuals were considered smokers if they smoked ≥7 cigarettes/week [75, 76].

^d^Only for smokers.

^e^Average use during the current consumption period.

^f^Cocaine_total_ (= cocaine + benzoylecgonine + norcocaine) as a more robust parameter [56].

^g^*χ*^2^ test for frequency data.

^h^Independent *t* test.

^i^Welch’s *t* test.

^j^Mann–Whitney *U*-test.

### Baseline (*T*_0_)

CU and HC did not differ in baseline cortisol and noradrenaline and subjective stress ratings (*p*s > 0.086; Table S2 for means and standard deviations). However, CU (*M* = 3.54, *SD* = 0.51, *n* = 42) had lower baseline ACTH levels than HC (*M* = 3.88, *SD* = 0.59, *n* = 36) (*t*(76) = 2.77, *p* < 0.01, Cohen’s *d* = 0.63).

### TSST

All analyses controlled for baseline (*T*_0_) levels of the respective dependent variable.

#### Noradrenaline

**Fig. 2:**
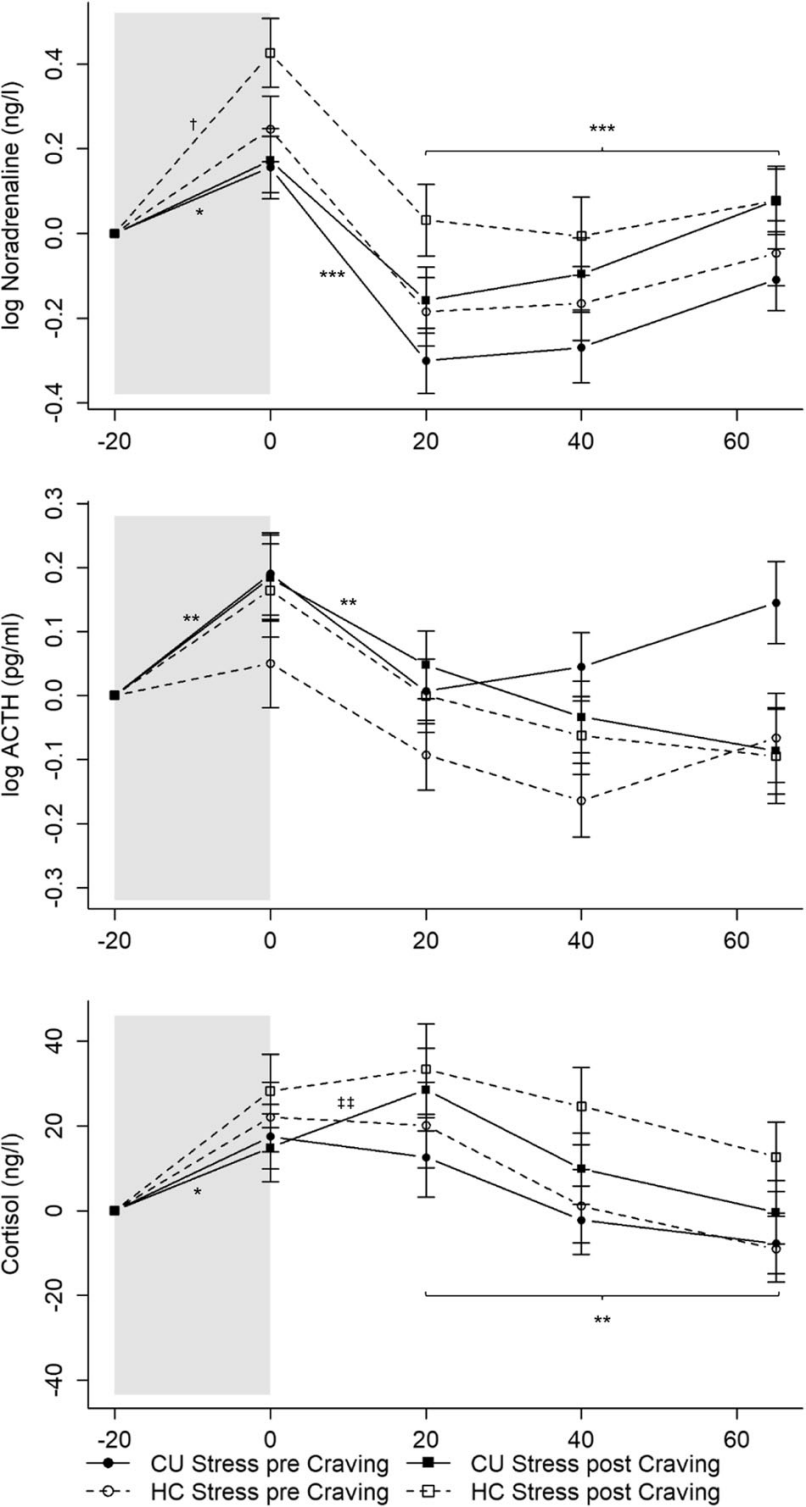
Mean levels and standard errors of the mean for noradrenaline, ACTH, and cortisol during the TSST. Gray shaded areas indicate the TSST. Values were normalized by subtracting the stress levels measured at *T*_1_ (−20 min) from all other values to facilitate interpretation of the stress reaction independently of effects of the circadian rhythm or baseline differences (*T*_0_). Cocaine users and healthy controls showed similar and significant increases in cortisol, ACTH, and noradrenaline with cocaine users undergoing the TSST after the Cocaine-Cue-Video demonstrating a stronger cortisol reaction. CU Cocaine users, HC Healthy controls. CU Stress pre Craving: *p < 0.05; **p < 0.01; ***p < 0.001; CU Stress pre Craving vs. HC Stress post Craving: ^†^*p* < 0.05; CU Stress pre Craving vs. CU Stress post Craving: ^‡‡^*p* < 0.01. CU Stress pre Craving: *n* = 22; CU Stress post Craving: *n* = 20; HC Stress pre Craving: *n* = 19; HC Stress post Craving: *n* = 17.

The Stress-pre-Craving CU’s noradrenaline levels significantly increased in response to the TSST (reactivity: *b* = 0.08, *t*(296) = 2.11, *p* < 0.05; Fig. 2). Subsequently, their noradrenaline levels significantly decreased until 20 min after the TSST (recovery_1: *b* = −0.24, *t*(296) = −8.71, *p* < 0.001) before slightly increasing again until 65 min later (recovery_2: *b* = 0.04, *t*(296) = 3.40, *p* < 0.001). Interactions between time components and Stress-post-Craving CU and Stress-pre-Craving HC were not significant (*p*s > 0.096), indicating that their noradrenaline trajectory was not significantly different from the Stress-pre-Craving CU. The Stress-post-Craving HC had a greater increase in noradrenaline levels during the TSST (*b* = 0.13, *t*(296) = 2.34, *p* < 0.05) compared to the Stress-pre-Craving CU. However, the HC-Stress-post-Craving*recovery_1 and HC-Stress-post-Craving*recovery_2 interactions were not significant (*p*s > 0.093).

#### ACTH

The Stress-pre-Craving CU’s ACTH levels increased in response to the TSST (reactivity: *b* = 0.09, *t*(296) = 3.20, *p* < 0.01; Fig. 2), immediately decreased until 20 min after (recovery_1: *b* = −0.09, *t*(296) = −3.09, *p* < 0.01), and remained stable until 65 min later (*p*s > 0.093). The interactions between time components and group were not significant (*p*s > 0.077). Thus, the ACTH response’s trajectory was not significantly different for the other groups.

#### Cortisol

Cortisol levels increased in response to the TSST for the Stress-pre-Craving CU (reactivity_1: *b* = 8.74, *t*(300) = 2.30, *p* < 0.05; Fig. 2) and stayed elevated until 20 min after the TSST (reactivity_2: *b* = −3.50, *t*(300) = −1.36, *p* = 0.176), followed by a decrease in cortisol levels until 65 min later (recovery: *b* = −4.42, *t*(300) = −3.17, *p* < 0.01). The interactions between time components and group did not become significant (*p*s > 0.117) except for a significant CU-Stress-post-Craving*reactivity_2 interaction (*b* = 9.26, *t*(300) = 2.47, *p* < 0.05). Overall, the cortisol response’s trajectory was not significantly different for the Stress-pre-Craving HC and the Stress-post-Craving CU and HC. However, the Stress-post-Craving CU showed a further increase in cortisol levels from *T*_2_ to *T*_3_ compared to the Stress-pre-Craving CU, demonstrating a stronger cortisol reaction for CU that experienced the TSST after the Cocaine-Cue-Video. Due to cortisol’s known circadian rhythm, the cortisol levels at *T*_1_ were estimated to be lower for the Stress-post-Craving HC (*b* = −21.77, *t*(73) = −2.66, *p* < 0.01) and CU (only marginally significant: *b* = −14.39, *t*(73) = −1.84, *p* = 0.070).

#### Stress and craving ratings

**Fig. 3:**
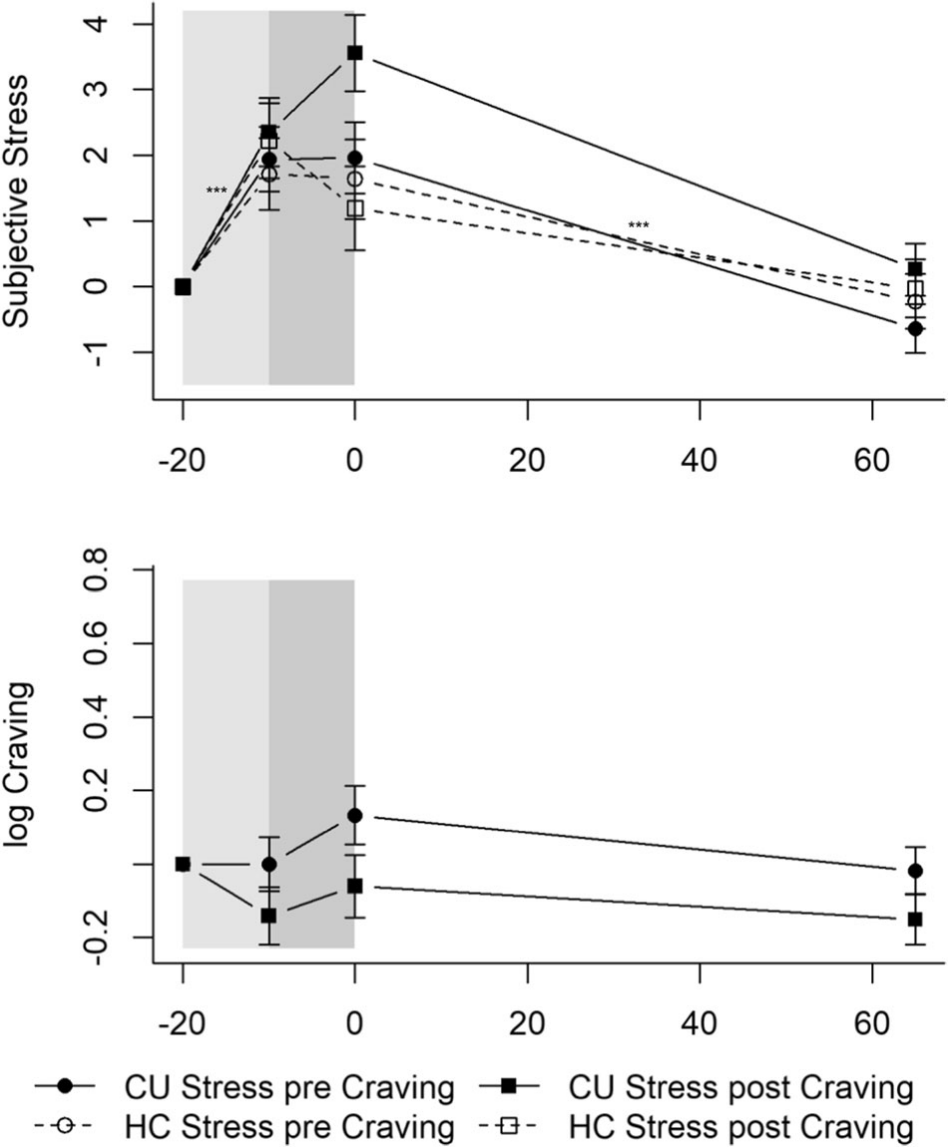
Mean levels and standard errors of the mean for subjective stress and craving during the TSST. Gray shaded areas indicate the TSST. Values were normalized by subtracting the levels measured at *T*_1_ (−20 min) from all other values to facilitate interpretation of the stress and craving reaction independently of effects of the circadian rhythm or baseline differences (*T*_0_). Cocaine users and healthy controls showed similar and significant increases in subjective stress, whereas the TSST did not induce craving in cocaine users. CU Cocaine users, HC Healthy controls. CU Stress pre Craving: ***p < 0.001. CU Stress pre Craving *n* = 25; CU Stress post Craving *n* = 22; HC Stress pre Craving *n* = 20; HC Stress post Craving *n* = 18.

The Stress-pre-Craving CU experienced greater subjective stress after the preparation period (TSST_preparation: *b* = 1.94, *t*(243) = 3.90, *p* < 0.001; Fig. 3). Their subjective stress ratings remained elevated until after the test period (reactivity: *b* = 0.02, *t*(243) = 0.04, *p* = 0.968) and decreased 65 min later (recovery: *b* = −2.60, *t*(243) = −5.21, *p* < 0.001). Interactions between time components and group were not significant (*p*s > 0.075). Thus, CU and HC of the other groups rated their subjective stress levels similarly during and after the TSST. Stress-post-Craving CU (*b* = −1.67, *t*(80) = −2.66, *p* < 0.01) and HC (*b* = −1.44, *t*(80) = −2.16, *p* < 0.05) estimated their subjective stress level at *T*_1_ to be lower than the Stress-pre-Craving CU.

Regarding craving within CU, the Stress-pre-Craving CU did not estimate their craving differently after the preparation period (TSST_preparation: *b* = −0.00, *t*(135) = −0.00, *p* = 0.997; Fig. 3) or directly after the test period (reactivity: *b* = 0.13, *t*(135) = 1.38, *p* = 0.170) or 65 min later (recovery: *b* = −0.15, *t*(135) = −1.56, *p* = 0.122). Interactions between time components and group did not become significant (*p*s > 0.322), indicating that the Stress-post-Craving CU were not different from the Stress-pre-Craving CU.

No significant group or order differences after the TSST were found for noradrenaline, ACTH, cortisol, and craving AUC_G_ in ANCOVAs controlling for the respective baseline levels (*p*s > 0.108; Table S3). Regarding subjective stress AUC_G_, an ANCOVA controlling for baseline subjective stress revealed a significant main effect for group (*p* < 0.05; Table S3). CU had a greater subjective stress AUC_G_ than HC over both levels of order.

#### Interaction of subjective and physiological stress responses

Three-way mixed ANOVAs showed a significant interaction between type-of-stress-response and group for cortisol (type-of-stress-response * group: *F*(1,74) = 4.45, *p* < 0.05) and ACTH (type-of-stress-response * group: *F*(1,74) = 7.32, *p* < 0.01) but not for noradrenaline (type-of-stress-response * group: *F*(1,73) = 0.47, *p* = 0.497). Sidak-corrected post hoc tests revealed that CU had a blunted HPA-axis response in contrast to their subjective stress response (refer to Figs. S1 and S2 for details).

### Cocaine-Cue-Video

All analyses controlled for baseline (*T*_0_) levels of the respective dependent variable. In the analysis of the noradrenaline trajectory, cannabis grams/week was additionally included.

#### Subjective stress and craving

**Fig. 4:**
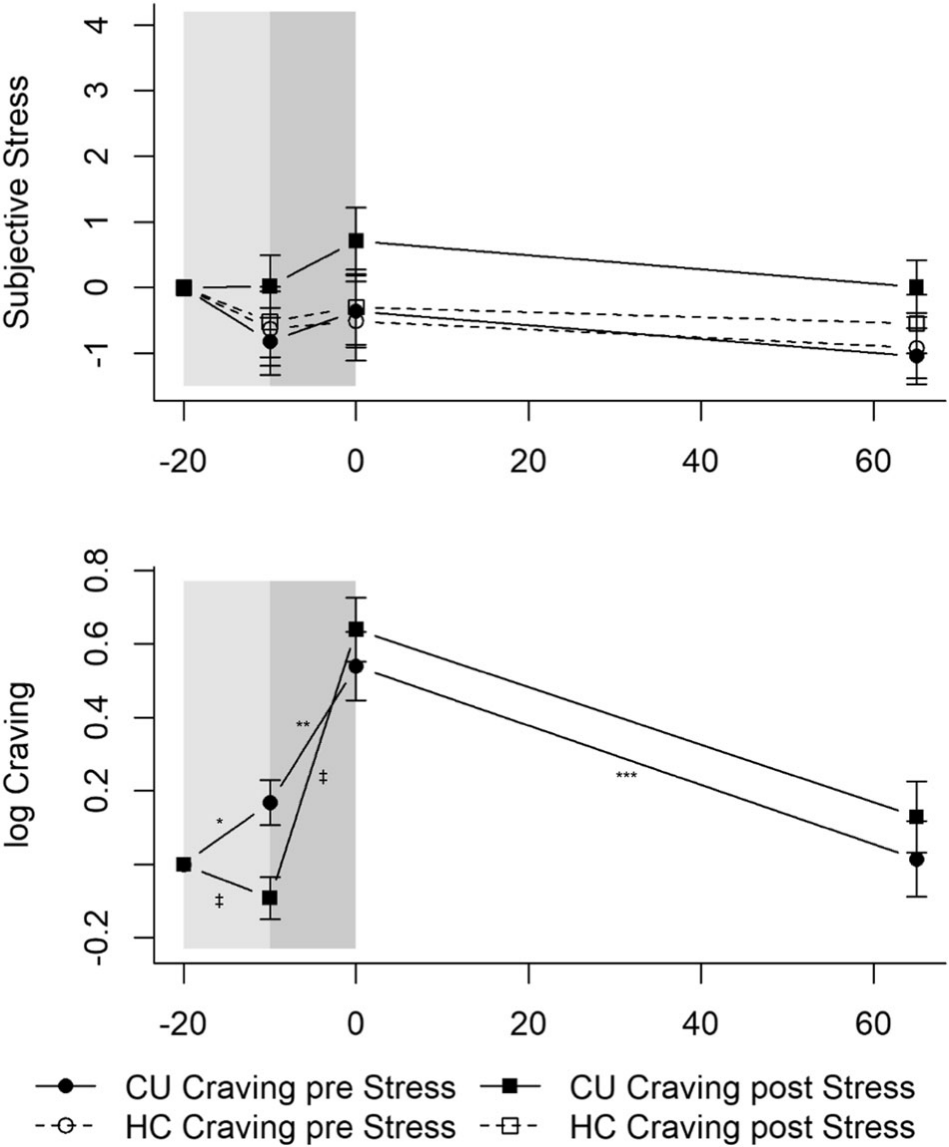
Mean levels and standard errors of the mean for subjective stress and craving during the Cocaine-Cue-Video. Gray shaded areas indicate the Cocaine-Cue-Video. Values were normalized by subtracting the levels measured at *T*_1_ (−20 min) from all other values to facilitate interpretation of the stress and craving reaction independently of effects of the circadian rhythm or baseline differences (*T*_0_). The Cocaine-Cue-Video did not induce a subjective stress response but elicited craving with cocaine users that underwent the Cocaine-Cue-Video after the TSST showing an enhanced craving response. CU Cocaine users, HC Healthy controls. CU Craving pre Stress: *p < 0.05; **p < 0.01; ***p < 0.001; CU Craving pre Stress vs. CU Craving post Stress: ^‡^*p* < 0.05. CU Craving pre Stress: *n* = 22; CU Craving post Stress: *n* = 25; HC Craving pre Stress: *n* = 18; HC Craving post Stress: *n* = 20.

Subjective stress ratings did not change significantly over the course of the Cocaine-Cue-Video for the Craving-pre-Stress CU (*p*s > 0.084; Fig. 4). No significant interactions between time components and group arose (*p*s > 0.197). The trajectory of the subjective stress ratings did not significantly change in the other groups.

As expected, craving increased in the Craving-pre-Stress CU during the Neutral-Video (Neutral-Video: *b* = 0.17, *t*(135) = 2.09, *p* < 0.05; Fig. 4) and kept increasing at a steeper rate during the Cocaine-Video (Cocaine-Video: *b* = 0.37, *t*(135) = 3.06, *p* < 0.01). It decreased until 65 min later (recovery: *b* = −0.52, *t*(135) = −3.77, *p* < 0.001). Significant Neutral-Video * CU-Craving-post-Stress (*b* = −0.26, *t*(135) = −2.36, *p* < 0.05) and Cocaine-Video * CU-Craving-post-Stress (*b* = 0.36, *t*(135) = 2.16, *p* < 0.05) interactions emerged. The Craving-post-Stress CU experienced a slight decrease in craving during the Neutral-Video and, subsequently, an even steeper increase in craving during the Cocaine-Video than the Craving-pre-Stress CU, indicating an enhanced craving response during the Cocaine-Video for CU that underwent the Cocaine-Cue-Video after the TSST.

#### Neuroendocrine response

**Fig. 5:**
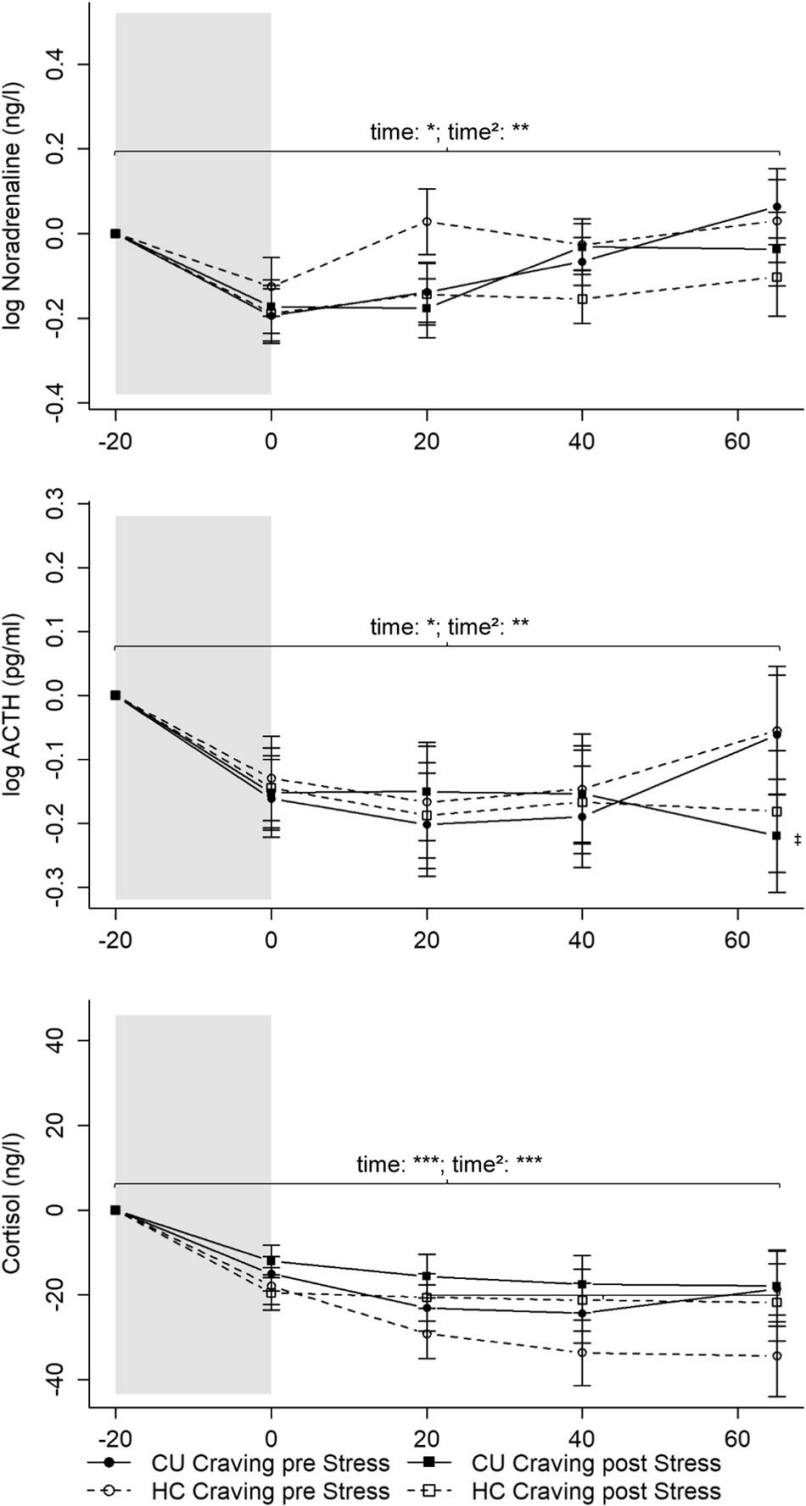
Mean levels and standard errors of the mean for noradrenaline, ACTH, and cortisol during the Cocaine-Cue-Video. Gray shaded areas indicate the Cocaine-Cue-Video. Values were normalized by subtracting the stress levels measured at *T*_1_ (−20 min) from all other values to facilitate interpretation of the stress reaction independently of effects of the circadian rhythm or baseline differences (*T*_0_). The Cocaine-Cue-Video did not induce a neuroendocrine stress response. CU = Cocaine users; HC = Healthy controls. CU Craving pre Stress: *p < 0.05; **p < 0.01; ***p < 0.001; CU Craving pre Stress time^2^ vs. CU Craving post Stress: ^‡^*p* < 0.05. Noradrenaline: CU Craving pre Stress: *n* = 20; CU Craving post Stress: *n* = 21; HC Craving pre Stress: *n* = 17; HC Craving post Stress: *n* = 19. ACTH and cortisol: CU Craving pre Stress: *n* = 20; CU Craving post Stress: *n* = 22; HC Craving pre Stress: *n* = 17; HC Craving post Stress: *n* = 19.

Contrary to psychosocial stress, the Cocaine-Cue-Video did not elicit a neuroendocrine stress response (Fig. 5). Noradrenaline (time: *b* = −0.06, *t*(298) = −2.30, *p* < 0.05; time^2^: *b* = 0.01, *t*(298) = 3.72, *p* < 0.01), ACTH (time: *b* = −0.09, *t*(304) = −3.94, *p* < 0.001; time^2^: *b* = 0.01, *t*(304) = 3.84, *p* < 0.001), and cortisol (time: *b* = −8.82, *t*(304) = −4.81, *p* < 0.001; time^2^: *b* = 0.79, *t*(304) = 4.84, *p* < 0.001) followed a curvilinear descent, with slight increases at the end of the test-session in the Craving-pre-Stress CU. No significant interactions between time components and group occurred for noradrenaline or cortisol (*p*s > 0.063). A significant time^2^ * CU-Craving-post-Stress interaction arose for ACTH (*b* = −0.01, *t*(304) = −2.03, *p* < 0.05), indicating that these CU did not experience increased ACTH levels at the end of the test-session. No further differences occurred for ACTH (*p*s > 0.140).

No significant group or order differences after the Cocaine-Cue-Video were found for AUC_G_ (*p*s > 0.058; Table S3).

All results remained robust against the influence of sex, age, BMI, verbal IQ, smoker, cannabis, MDMA, and alcohol grams/week. To facilitate the interpretation of the TSST and Cocaine-Cue-Video response, we normalized all the values by subtracting the stress levels at *T*_1_ (−20 min). The trajectories with normalized values can be seen in Figs. 2–5 (non-normalized values can be seen in Tables S4 and S5); the random-effect variances can be seen in Tables S6 and S7.

### Correlational analyses

AUC_G_ for noradrenaline, ACTH, cortisol, and subjective stress were not associated with total hair concentrations of cocaine metabolites [56] (cocaine_total_) within CU only (*r*_s_s < 0.103, *p*s > 0.377). Cocaine_total_ was, however, positively associated with baseline craving (*r*_s_ = 0.634, *p* < 0.001, *n* = 47) and with craving AUC_G_ in the Cocaine-Cue-Video (*r*_s_ = 0.463, *p* < 0.001, *n* = 47). For the TSST, this was only marginally significant (*r*_s_ = 0.314, *p* = 0.032, *n* = 47). In general, greater cocaine consumption was associated with greater craving.

## Discussion

This study investigated the effect of acute psychosocial stress, cocaine-cue reactivity, and their interactions on psychophysiological stress responses in chronic CU. This study’s innovation lies in its subsequent application of psychosocial stress and cocaine-cue-based craving induction in a randomized, crossed, and balanced order. Thus, half of the participants received the psychosocial stressor first, and the other half received the craving induction first. Unlike previous studies (e.g., [24, 25]), dysregulated acute stress responses to experimentally-induced psychosocial stress in CU were not observed. Though baseline cortisol and noradrenaline levels were normal in CU, their baseline ACTH level, compared to HC, was significantly lower. CU experienced strong craving but no measurable neuroendocrine stress response during the cocaine video cue. Most importantly, the cortisol response was enhanced by previous craving, while craving was intensified by a preceding confrontation with the psychosocial stressor. Directly contrasting their subjective stress response, CU had a blunted HPA-axis response.

### Psychosocial stress

In response to the psychosocial stressor, when the order of the stress/craving induction was not considered, CU and HC showed similar and significant increases in cortisol, ACTH, noradrenaline, and subjective stress. Hence, contrary to our hypothesis, CU did not experience a blunted HPA-axis response as shown in some past studies (e.g., [24, 25]) when their subjective response was not included in the analysis. However, our results are in line with Moran-Santa Maria et al. [57], who observed no differences in the plasma cortisol and ACTH response to the TSST between HC and CU. In line with this, no general differences in the HPA-axis reactivity between CU and HC were seen in a study that used corticotropin-releasing hormone infusion as a stress challenge [58]. Waldrop et al. [25] identified a blunted plasma cortisol response during the TSST only in female CU. Compared to male CU, our study’s female CU also had a blunted cortisol response during the TSST (Table S8). Waldrop et al. [25] observed no ACTH differences. As mentioned previously, most studies that employed the TSST or personalized stress imagery to assess the HPA-axis response in CU did not include a control group and used pre-post comparisons or a neutral condition to compare physiological stress responses: Harris et al. [24] used pre-post comparisons in the TSST and personalized stress imagery to determine blunted salivary cortisol responses in CU and methamphetamine users, while Sinha et al. [28–30] and Fox et al. [32] assessed the physiological stress response in personalized stress imagery using a neutral condition. Sinha et al. [28, 29] observed positive change scores from baseline, and therefore slight increases in salivary cortisol following stress, compared to a neutral condition. Although Sinha et al. [30] and Fox et al. [32] demonstrated increased plasma cortisol and ACTH levels compared to a neutral condition, the change scores from baseline were negative for plasma cortisol in Sinha et al. [30] and for plasma cortisol and ACTH in Fox et al. [32]. These results indicate an attenuation of the diurnal cortisol and ACTH decrease and could thus also be interpreted as a blunted HPA-axis response. Overall, the past results are heterogeneous.

One reason no clear differences in the psychosocial stress reactivity between CU and HC were found might be that our CU seemed to be relatively high-functioning individuals, with only 19% (*n* = 9) being unemployed at screening. Thus, despite their regular cocaine use and CUD, most of them could manage their daily lives. Another possible explanation might be that HPA-axis responses in CU normalized due to prolonged abstinence. At the time of the stress assessment, CU could be either abstinent (<6 months at screening) or on-going users. On average, our sample was abstinent for 26.3 days before the stress assessment, used 4 grams of cocaine per week, and had relatively high cocaine hair concentrations (cocaine_total_: 24,703 pg/mg; compare to Table 1). We assume that possible cocaine-induced adaptations of the stress system are reversible if cocaine use decreases/ceases. However, due to the relatively short abstinence duration and the relatively high cocaine consumption of our sample before the stress assessment, we would not expect normalization of HPA-axis responses so soon. Similarly, cognitive deficits in CU improved if cocaine use decreased which was particularly pronounced in CU with sustained abstinence for at least 6 months prior to the cognitive assessment [59]. Interestingly, CU showed a downregulated baseline ACTH tone. In previous studies, lower ACTH levels were also found in heavy drinkers [60] and individuals at risk of developing alcohol use disorder [61]. However, including self-reported alcohol grams/week did not change this study’s results. Since cocaine administration acutely stimulates ACTH secretion [10, 62], the blunted ACTH tone could be due to repeated cocaine consumption, which led to HPA-axis adaptations. Similarly, chronic CU’s downregulated NR3C1 expression was suggested to be caused by the excessive stimulation of cortisol secretion through cocaine consumption [21, 22]. However, possible cocaine-related HPA-axis adaptations did not seem to have affected cortisol and CU’s physiological stress responses. Baseline cortisol levels might not have been affected due to separate activation of the adrenal glands via the sympathetic nervous system. Accordingly, baseline noradrenaline levels, a measure of SAM-axis activity, were also not affected in CU.

During the TSST, CU had a greater subjective stress AUC_G_ than HC. This is in line with Waldrop et al. [25], who observed greater subjective stress in CU than HC, and Moran-Santa Maria et al. [57], who found the greatest subjective stress in CU with early-life stress.

Surprisingly, the TSST did not induce robust craving symptoms. Previous studies reported increased cocaine craving after personalized stress imagery [28–30] or the TSST [25, 57]. However, craving did not increase after a standard speech stress task [28] or in another study using the TSST [63]. Interestingly, Fox et al. [32] observed greater craving only in high-frequency and not in low-frequency alcohol and CU after personalized stress imagery. Thus, a craving response to stress may depend on cocaine use intensity, comorbidity, and stress modality. Based on the drug use information in Fox et al. [32], this study’s CU seem to be situated between the high- and low-frequency users of their study. Therefore, present CU might have a lower cocaine use intensity compared to Fox et al. [32], so they did not show a measurable craving response during the TSST. However, comparing our CU after categorizing them into light- (<5000 pg/mg) and heavy-consumption CU (≥ 5000 pg/mg) according to cocaine hair concentrations, no significant differences regarding the craving response, besides stronger craving in heavy CU, were observed (Table S9). However, the groups were small (*n*s = 6–16), so this needs to be interpreted with caution. Furthermore, the TSST could not have been a relevant craving-inductor to our CU. The TSST uses high levels of social-evaluative threat and uncontrollability specifically qualified to evoke HPA-axis reactivity in a majority of individuals [41, 42], but such stressful situations are not common. Guided stress imagery, however, can be tailored to an individual’s own stressful experiences [28]. Thus, the TSST and personalized guided stress imagery likely elicit different affective stress responses [36] that, in the case of the TSST, might not be associated with cocaine use as a coping mechanism.

### Cocaine-Cue reactivity

In line with Sinha et al. [29, 30] and Waldrop et al. [25], the Cocaine-Cue-Video, as shown before in a different sample [44], elicited a strong craving response in CU. Remarkably, the cocaine-cue paradigm did not induce a neuroendocrine stress response in CU or HC. This goes against our expectations and results from other studies that used drug-cue imagery, which increases cortisol, ACTH, and noradrenaline [29, 30]. Moreover, the Cocaine-Cue-Video did not induce a clear subjective stress response. Using in vivo cocaine-cues and a cocaine-cue video, Waldrop et al. [25] reported greater subjective stress increases in CU than HC.

The lack of a neuroendocrine stress response in our CU in response to the cue paradigm might be because, unlike Sinha et al. [29, 30], we did not use drug-cue imagery based on a personalized script with a recent cocaine-related situation that caused subsequent cocaine use, which might have identified more relevant cocaine-cues that could have induced a neuroendocrine stress response. Nevertheless, our Cocaine-Cue-Video robustly evoked craving, ensuring the task’s internal and ecological validity. Furthermore, Fox et al. [32] observed that only high-frequency CU showed a cortisol and ACTH response to drug-cue imagery. As mentioned before, our CU seem to be situated in between Fox et al.’s samples [32] regarding their cocaine consumption, which possibly explains the absent neuroendocrine response. However, comparing light and heavy CU in our study regarding their neuroendocrine stress response to the Cocaine-Cue-Video did not reveal significant differences (Table S10). Moreover, unlike our study, most of the past studies investigated treatment-seeking CU. For treatment-seeking individuals, experiencing a cue paradigm could be more stressful, especially if they aim to stay abstinent [30]. Only 32% (*n* = 15) of our CU reported being in treatment or counseling for cocaine use; 15% (*n* = 7) reported being in treatment or counseling for other mental problems, so 53% (*n* = 25) of them were not treated. Accordingly, only 45% (*n* = 21) claimed they wanted to entirely quit cocaine consumption, which might explain the lack of an HPA-axis activation during or after the cocaine-cue paradigm.

### Interaction between psychosocial stress and craving

#### Cocaine-Cue-Video first, TSST second

We observed a stronger cortisol reaction in CU who took the TSST after completing the cocaine-cue paradigm. Cortisol further increased right after completing the TSST (*T*_2_) until 20 min after (*T*_3_) compared to the CU who completed the TSST at the beginning of the test-session (Fig. 2). The HC who completed the TSST after watching the Cocaine-Cue-Video did not have the same cortisol reaction. Based on the lack of HPA-/SAM-axis and subjective stress reactivity during the Cocaine-Cue-Video, the further increase observed in the CU who performed the TSST after the video was, we assumed, due to the craving they experienced beforehand. However, as the effect was only significant in cortisol and rather subtle, it should be considered preliminary and awaiting replication.

#### TSST first, Cocaine-Cue-Video second

CU who completed the cocaine-cue paradigm after the psychosocial stressor had first a decrease in craving during the neutral video and second a steeper increase in craving during the cue video than CU who completed the cocaine-cue paradigm at the beginning of the test-session. A comparable decrease in craving during the first half of the second stress challenge can also be observed in CU that underwent the TSST as the second stress challenge (compare to Fig. 3). Individuals underwent a short break right before the second stress challenge, which may have led to a decrease in estimated craving, as individuals were more relaxed. Estimated craving levels after the Cocaine-Cue-Video were similar between both groups but the craving reaction itself during the cue video seemed to be stronger in CU that underwent the Cocaine-Cue-Video after the TSST. However, similarly to the enhanced cortisol response during the psychosocial stressor, the effect is subtle and awaits replication. Nevertheless, our findings are align with Duncan et al. [39], who observed enhanced activation in brain areas associated with reward and conditioned cues during cocaine-cue imagery if a stressor was present. Moreover, personalized stress imagery decreased nicotine-deprived smokers’ capacity to resist smoking [64, 65], with greater cortisol, ACTH, and craving levels associated with decreased latency to smoke and increased smoking satisfaction and reward [64]. The results of our study and these studies [39, 64, 65] can be interpreted within the broader context of animal models. First, plasma corticosterone increases cocaine reward sensitivity and influences cue-induced reinstatement of cocaine-seeking (for a review, see [14, 15]). Second, stress-induced glucocorticoids enhance dopamine release in the mesocorticolimbic reward system (for a review, see [5]). The preceding TSST-induced cortisol release could thus have amplified the cue-induced dopamine-mediated prediction error for cocaine reward, and therefore craving, due to the preceding additional stimulation of the dopaminergic reward system through the TSST. Furthermore, instead of protective effects of oral cortisol administration on craving in low-dose heroin users [37], we found an augmenting effect. This difference might be explained by cocaine’s activating effect on the HPA-axis [7–9], while heroin’s effects are attenuating (for a review, see [6]).

Previous completion of the TSST did not influence the stress reaction during the cocaine-cue paradigm. This is in line with findings from prescription opioid users [33]. Moreover, the socially evaluated cold pressor stress test did not potentiate smoking-related drug-seeking behavior [35].

An alternative explanation as to why effects were only observed after two subsequent challenging paradigms in CU but not HC may be a stronger rise of ego depletion [66, 67] or mental fatigue (for a review see [68]). Considering that CU show cognitive deficits (e.g., [47]), greater trait impulsivity (e.g., [69, 70]), and lower inhibitory control (e.g., [71, 72]), it may not be surprising if two subsequent tasks, especially if the TSST was done first, were cognitively more demanding for them. This may have led to increased ego depletion/mental fatigue during the second task and the reported subtle effects may be their consequence. However, CU did not estimate their level of fatigue to be worse than HC before the second challenge independently of the order of the challenge and levels of fatigue did not change significantly from before the first to the second challenge for neither CU nor HC independently of the order (*p*s > 0.074).

### Limitations

Sample sizes for the subgroups were rather small. It is therefore conceivable that the power to detect differences between subgroups was not sufficient. Furthermore, half of our CU were currently in treatment, whereas the other half was not rendering our CU sample rather heterogeneous. However, in order to investigate dose-response relationships between cocaine use intensity and outcome variables, it is conducive to recruit a broader, more varied sample as too much homogeneity is not advantageous to investigate dose-response relationships. Nevertheless, our results should be considered as preliminary as we only observed few significant effects with small effect sizes respectively, which is why they should be replicated in larger, more homogeneous samples to improve generalizability of results. Furthermore, recent acute cocaine use may have affected psychosocial stress and cocaine-cue reactivity, which is why we may not have been able to observe altered responses in CU. Hence, this should be kept in mind while interpreting results. Although we report urine toxicology results in Table 1, a positive urine test does not allow us to draw conclusions on how recent cocaine use was. The inactive cocaine metabolite benzoylecgonine is detectable in urine for up to 4 days [73] and in heavy users even 3 weeks after cessation of use [74]. Thus, to control for acute effects of cocaine use, cocaine blood concentrations should have been assessed.

## Conclusion

Regular but high-functioning CU displayed neither dysregulated HPA-axis responses nor robust craving symptoms to experimentally-induced psychosocial stress. In contrast, the cocaine-cue paradigm solidly evoked craving but no neuroendocrine stress response. Our results indicate that psychosocial stress and craving interacted in CU. First, cortisol reactivity to the TSST was enhanced when the cocaine-cue preceded psychosocial stress. Second, cocaine-cue-induced craving was intensified when psychosocial stress preceded the cocaine-cue. Thus, stress and craving have mutually augmenting effects on HPA-axis reactivity and craving in CU, which possibly contribute to the maintenance of and relapse in chronic cocaine use. For instance, situations with potentiated HPA-axis reactivity or craving through previous exposure to environmental cocaine-cues or psychosocial stress may pose more at-risk situations for subsequent cocaine use. As the influence of stress and craving probably blend together in everyday life, this study’s results should have a greater validity for CUs’ daily lives compared to studies that investigated the influence of stress and craving separately. Baseline ACTH levels were lower in CU, nevertheless pointing to potential predispositions or cocaine-induced HPA-axis adaptations, which did not influence acute stress responses to our social stressor. However, dysregulations in the physiological stress response might arise later during addiction with continued cocaine use.

Our results extend the current knowledge in the field of stress and craving in CUD. Individuals with regular cocaine use do not necessarily show dysregulated HPA-axis activity in response to psychosocial stress or show HPA-axis reactivity to cue-induced craving, although these reactivity patterns may be associated with the negative reinforcement properties of cocaine use [2, 3, 5, 23, 25, 28–30]. However, the combination of stress and craving seem to impact relapse vulnerability and their interactions should thus be investigated in future studies and targeted in new treatment approaches.

## Supplementary information

Supplementary Material
